# Population Level Analysis of Evolved Mutations Underlying Improvements in Plant Hemicellulose and Cellulose Fermentation by *Clostridium phytofermentans*

**DOI:** 10.1371/journal.pone.0086731

**Published:** 2014-01-22

**Authors:** Supratim Mukherjee, Lynmarie K. Thompson, Stephen Godin, Wendy Schackwitz, Anna Lipzen, Joel Martin, Jeffrey L. Blanchard

**Affiliations:** 1 Department of Microbiobiology, University of Massachusetts, Amherst, Massachusetts, United States of America; 2 Department of Chemistry, University of Massachusetts, Amherst, Massachusetts, United States of America; 3 Biology Department, University of Massachusetts, Amherst, Massachusetts, United States of America; 4 Genomic Technologies, Joint Genome Institute, Walnut Creek, California, United States of America; Instituto de Tecnologica Química e Biológica, UNL, Portugal

## Abstract

**Background:**

The complexity of plant cell walls creates many challenges for microbial decomposition. *Clostridium phytofermentans*, an anaerobic bacterium isolated from forest soil, directly breaks down and utilizes many plant cell wall carbohydrates. The objective of this research is to understand constraints on rates of plant decomposition by *Clostridium phytofermentans* and identify molecular mechanisms that may overcome these limitations.

**Results:**

Experimental evolution via repeated serial transfers during exponential growth was used to select for *C. phytofermentans* genotypes that grow more rapidly on cellobiose, cellulose and xylan. To identify the underlying mutations an average of 13,600,000 paired-end reads were generated per population resulting in ∼300 fold coverage of each site in the genome. Mutations with allele frequencies of 5% or greater could be identified with statistical confidence. Many mutations are in carbohydrate-related genes including the promoter regions of glycoside hydrolases and amino acid substitutions in ABC transport proteins involved in carbohydrate uptake, signal transduction sensors that detect specific carbohydrates, proteins that affect the export of extracellular enzymes, and regulators of unknown specificity. Structural modeling of the ABC transporter complex proteins suggests that mutations in these genes may alter the recognition of carbohydrates by substrate-binding proteins and communication between the intercellular face of the transmembrane and the ATPase binding proteins.

**Conclusions:**

Experimental evolution was effective in identifying molecular constraints on the rate of hemicellulose and cellulose fermentation and selected for putative gain of function mutations that do not typically appear in traditional molecular genetic screens. The results reveal new strategies for evolving and engineering microorganisms for faster growth on plant carbohydrates.

## Background

The complexity of plant cell walls, in which cellulose microfibrils are linked via hemicellulosic tethers, further strengthened by associations with lignin, and embedded in the pectin matrix, present many challenges for microbial decomposition [1]–[4]. Cellulose, the primary component of cell walls, is an insoluble polysaccharide consisting of a long linear chain of β(1→4) linked D-glucose units. Cellulose is broken down into smaller oligodextrins such as cellobiose by extracellular fungal and bacterial enzymes. Hemicellulose is comprised of many different carbohydrates. Xylans frequently form the backbone of hemicellulose in many plant cell walls and are almost as ubiquitous as cellulose. Xylan is also cleaved into smaller oligomers, and the monosaccharide xylose by microbial enzymes.

Understanding rates of plant cell wall breakdown is of fundamental importance in many research areas. In ecology, the decomposition of the plant leaf litter has long been a central focus in determining rates of carbon cycling [5]–[8]. The “recalcitrance” of the plant cell wall in human and animal digestive systems decreases the amount of energy extracted from plants [9]–[12]. For example, less than half of carbohydrates in hay are extracted during passage through a ruminant and even less through other digestive systems [9]. In the development of bioproducts, such as biofuels, the primary challenge is to create more economical processes to saccharify plant cells walls into simple fermentable sugars [13]–[15].

The transport and metabolism of simple carbohydrates in bacteria has been extensively studied at the molecular level and the transport and regulatory mechanisms are well understood in several systems [16], [17]. Recently, experimental evolution has become an effective method to improve metabolic rates. These experiments are based in principle on controlled microbial laboratory evolution experiments initiated by Richard Lenski in 1988 using *Escherichia coli* grown on a defined medium [18]. In competition studies on substrates that use the same mechanism of transport as glucose, the evolved lines are generally more fit, which suggests that higher rates of glucose transport was an important target of selection [19]. Adaptive evolution of *S. cerevisiae* on glucose using prolonged chemostat cultivation resulted in the selection of mutants with one or more gene duplications in high-affinity hexose transporters [20]. Genome-wide transcriptome analysis revealed changes in the expression of many genes, including several genes encoding proteins involved in central carbon metabolism [21].

Since these initial studies, experimental evolution strategies using microorganisms have led to the selection of quantitative differences between strains and have become an important tool for improving selected phenotypes and the modification and optimization of microbial strains [22]–[27]. Recent developments in genomics and bioinformatics along with the ability to sequence entire bacterial genomes have played a significant role in developing the field of experimental evolution [28]–[36].

*Clostridium phytofermentans,* isolated from forest soil near the Quabbin Reservoir in Massachusetts, U.S.A., can grow directly on many types of plant litter, utilizing the cellulose, hemicellulose and pectin components [37]. Analysis of the genome sequence revealed the presence of more than a hundred glycoside hydrolases distributed across 38 families and detected over a hundred ATP binding cassette (ABC) transport systems, around 50% of which are dedicated to carbohydrate transport (Petit et al, submitted). Custom Affymetrix microarrays on 17 purified plant cell wall carbohydrates have elaborated that *C. phytofermentans* has a well-coordinated system to sense and differentially regulate carbohydrate degradation and transport (Petit et al, submitted). Quantitative proteomic analysis also detected large quantities of extracellular solute binding proteins responsible for transporting specific sugar molecules into the cell [38].

The innate ability of *C. phytofermentans* to grow on a wide range of substrates enabled us to apply experimental evolution as a tool to develop strains and populations with improved rates of hemicellulose and cellulose usage. This study applies a combination of automated and manual population-level analysis techniques to identify mutations. Our results reveal new strategies for adapting and engineering microorganisms by identifying mutations that alleviate constraints on plant cell wall carbohydrate breakdown.

## Results

### Growth and Product Measurements

**Figure 1:**
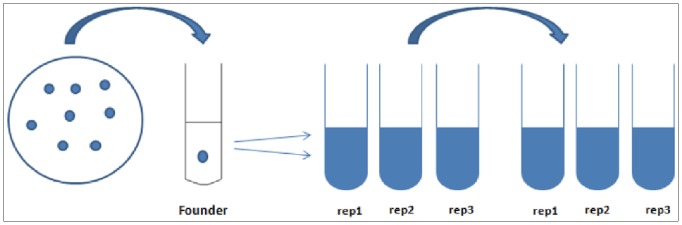
Schematic representation of the adaptive evolution process starting from an isogenic founder. Rep 1, 2 & 3 represent the three replicates in each of the three individual lines.

**Figure 2:**
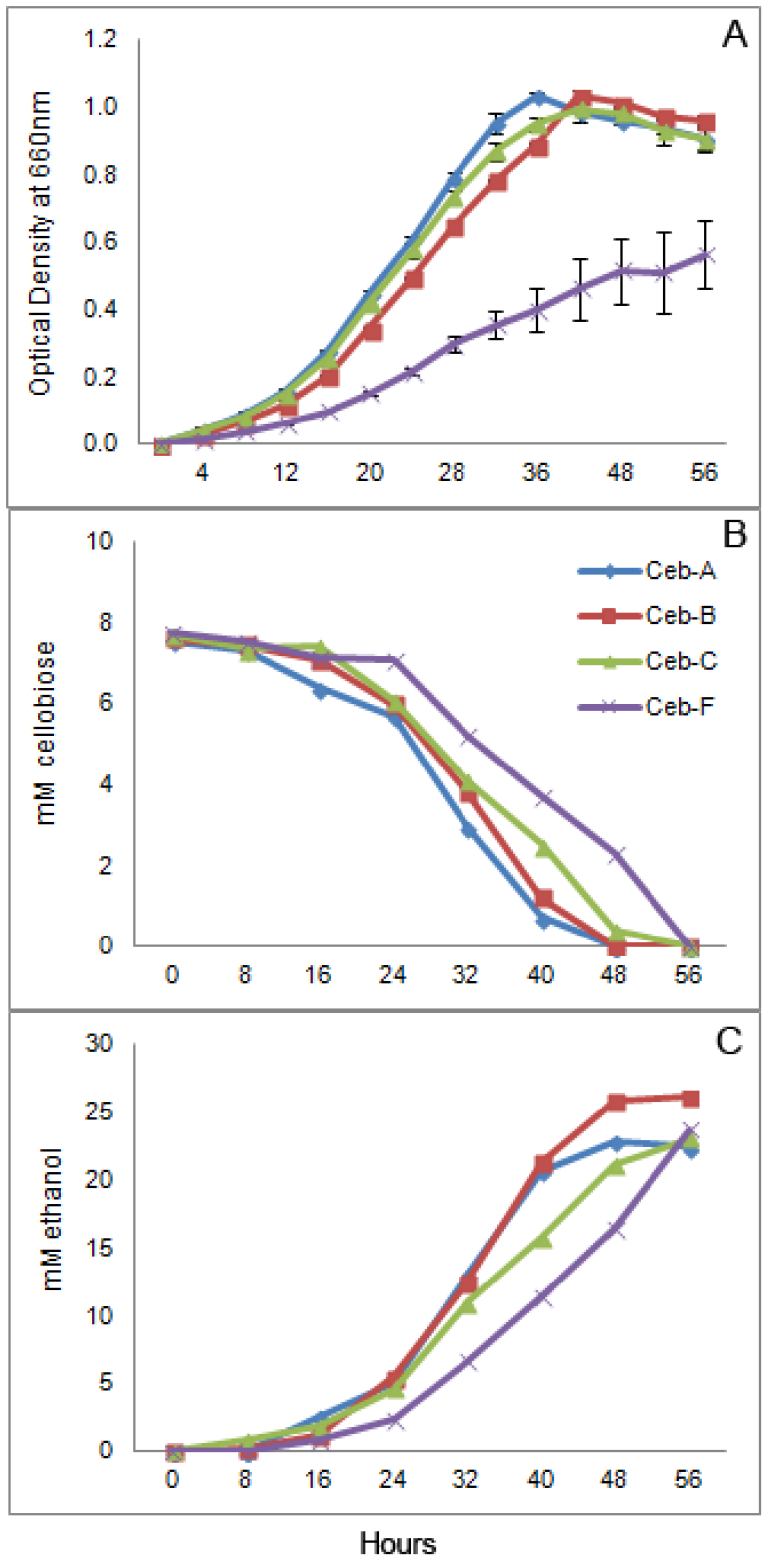
Growth, cellobiose utilization and ethanol production of cellobiose adapted populations and the founder. Growth (A) was measured every four hours as change in optical density in a spectrophotometer. Supernatant was collected every eight hours for measuring cellobiose utilization (B) and ethanol production (C) rates. Cellobiose and ethanol values represent an average of two independent samples.

**Figure 3:**
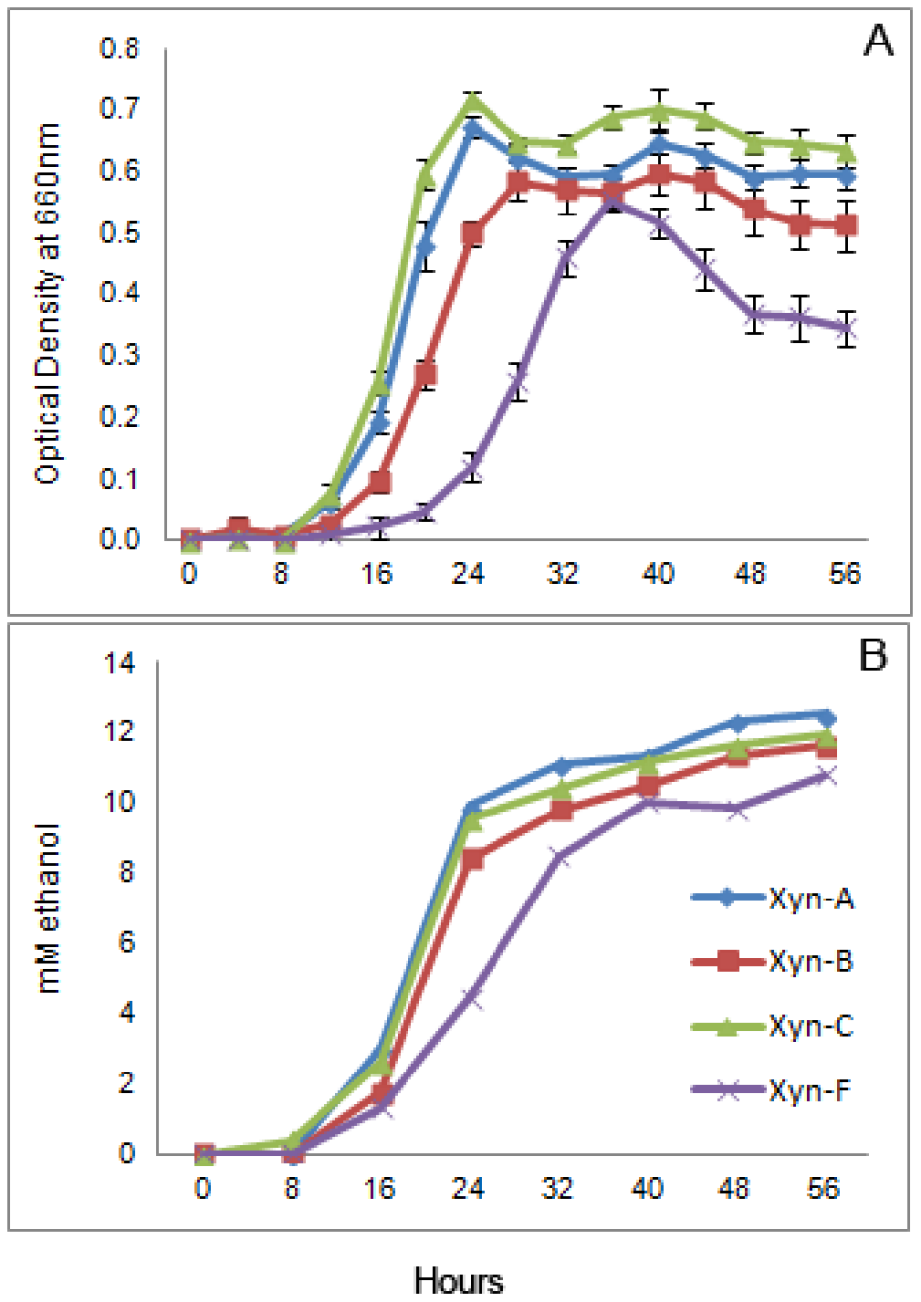
Growth and ethanol production of xylan-adapted populations and the founder. Growth (A) was measured every four hours as change in optical density in a spectrophotometer. Supernatant was collected every eight hours for measuring ethanol production (B) rates. Ethanol values are an average of two independent samples.

Serial transfers of three parallel lines from the founder were conducted in the following manner: every 24 hours for 60 days on xylan, every 48 hours for 60 days on cellobiose and every 7 days for 50 weeks on cellulose (Fig. 1). Growth measurements of cellobiose-adapted populations showed substantial improvements in terms of decreased lag phase and higher growth rates during early exponential growth (Fig. 2A). The mean generation times during log phase growth of Ceb-A, Ceb-B and Ceb-C were 2.79, 2.63 and 2.83 hours respectively compared to that of 4.48 hours for the founder, Ceb-F. Similar results were obtained for xylan-adapted lines, which had mean generation times of 0.75, 0.83 and 0.55 hours for Xyn-A, Xyn-B and Xyn-C respectively compared to 1.06 hours for the founder Xyn-F during log phase growth (Fig. 3A).

During growth on cellobiose, cellulose and xylan as substrates, *C. phytofermentans* produces ethanol and acetate as major liquid fermentation products along with a small amount of lactate and formate [37]. Improvements in growth rate for cellobiose and xylan evolved lines resulted in corresponding increases in ethanol production rates during early to mid-exponential growth (Fig. 2C & 3B).

**Figure 4:**
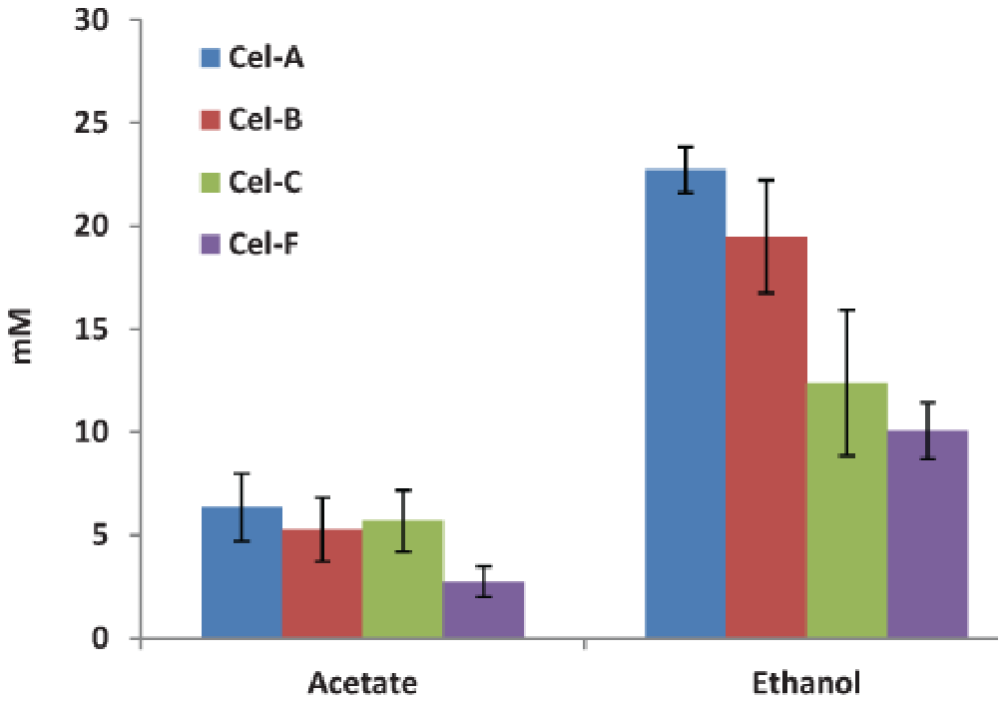
Major fermentation product formation by cellulose adapted populations and founder after 10 days of growth.

Because cellulose is insoluble and interferes with absorbance measurements, optical density could not be used as a measure of growth on cellulose. Since growth rate was shown to be directly linked to fermentation product formation in cellobiose and xylan evolved lines, we used product formation as a proxy for growth in our cellulose evolved lines (Fig. 4). In all lines ethanol was observed to accumulate at faster rates.

### Whole Genome Sequencing and Analysis of Evolved Populations

We extracted and sequenced DNA directly from a sample of the population, thus capturing variation that would be missed by sequencing an isolate. An average of 13,600,000 paired end reads were generated per population resulting in ∼300 fold coverage of each site in the genome. The reads were aligned to the reference *C. phytofermentans* genome (NC_010001) and putative SNPs and small indels were called using maq-0.7.1 [39] at default values and using the script SNPdetection_pooledSequence with a haplotype number of 10 [40]. Since the Holt script does not attempt to identify indels, a rough estimate of the allele frequency was calculated by counting the number that showed the indel vs the number that did not show the indel as reported by Maq. Indels supported by only a few reads were considered to be false positives. Putative large structural variants were called using BreakDancer [41], which has been specifically designed for analyses with paired end reads. Instances of false positive rates are known to be high with BreakDancer, especially for sequences where the read length is greater than the read depth. However, since our read depth on average was 2.5 times that of the read length, we expected the error rates to be low. In addition, all the large structural variations were corroborated by manual inspection to reduce the rate of false positives and to determine the exact coordinates of the break points.

The populations were further analyzed to detect SNPs at a minimum frequency of 5% and allele frequency estimates were calculated [40]. The variant calls of the founder facilitated mutation identification in the adapted lines with high confidence. The effectiveness of this method to analyze population samples and its rate of false positive and false negative prediction was evaluated by simulating a population by pooling and comparing reads from closely related *E. coli* strains with known variants (data not shown). In general, for sequencing depth of around 250x both Holt’s script and Maq had a very low false positive rate while MAQ had a slightly higher rate of false negatives, especially for SNPs predicted to be present at a very low frequency. To reduce the error rate in our analysis, the SNPs predicted using Holt’s script and MAQ were compared and validated manually. A SNP called by either of these programs was considered to be a false positive if many strand biased G/C mismatches were observed near the putative SNP (suggesting a sequence specific error), if the putative SNP was never detected in the middle of a read (suggesting an alignment problem), or if there was a signature of a large insertion or deletion in the region of a putative SNP. SNP allele frequency accuracy was determined by Holt’s script and was corroborated using qPCR (Battista, personal communication). Greater than 90% of the allele frequency estimates were observed to agree with qPCR estimates. A summary of the sequencing results is shown in Table 1. The complete list of mutations identified in the adapted populations including the type of change, genomic position and the predicted function of the effected gene is displayed in Table S1.

**Table 1:** Summary of sequencing results including the average read-depth, number of reads and mutations detected in the adapted populations as well as in the founder line.

Population	Average ReadDepth	Number of pairedend reads	Number of putativeSNPs	Number of putativeindels	Sum
**Founder**	285.84	14,059,906	3	1	4
**Ceb-A**	266.92	13,143,086	3	1	4
**Ceb-B**	276.43	13,588,562	0	3	3
**Ceb-C**	285.31	14,017,984	1	3	4
**Xyn-A**	276.77	13,595,790	2	2	4
**Xyn-B**	264.53	13,000,804	4	3	7
**Xyn-C**	291.32	14,320,866	2	2	4
**Cel-A**	261.96	12,976,078	8	5	13
**Cel-B**	286.16	14,070,080	3	10	13
**Cel-C**	278.56	13,737,052	7	11	18

**Figure 5:**
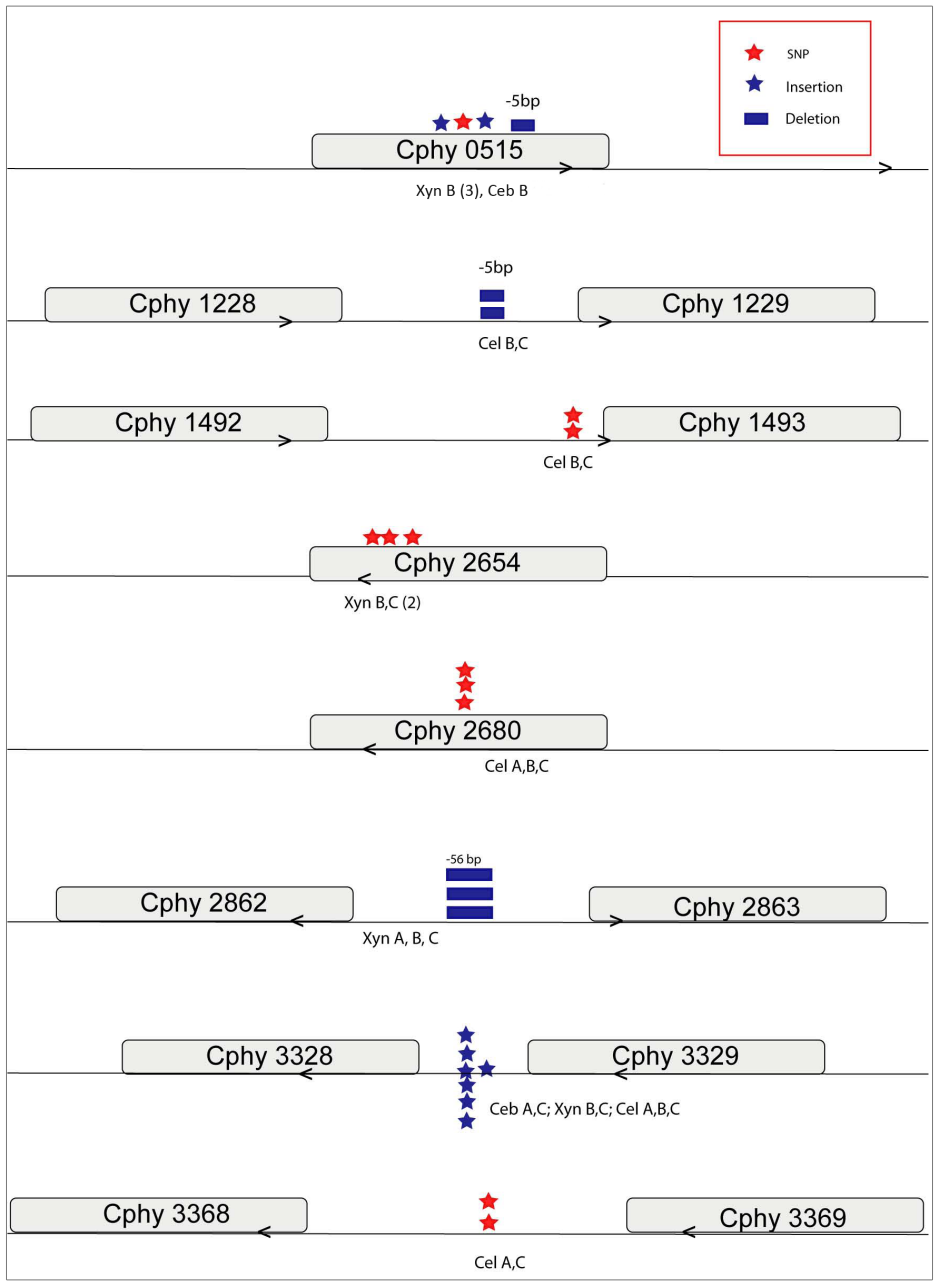
Genes and intergenic regions where multiple mutations were detected. Mutation hotspots which were identified in multiple evolved lines or in the same population more than once. For example, Cphy 0515 was observed to have a separate SNP (red star), insertion (blue star) and deletion (blue box) in Xyn-B and one insertion in Ceb-B (See Table S1).

Mutations which were predicted to be present in multiple lines, as well as genes and intergenic regions harboring several mutations are depicted in Figure 5. The cellulose-adapted lines, especially Cel-B and Cel-C show a high level of insertion sequence (IS) element activity (Table S1). A large number of mutations were detected in non-coding regions of the genome. In the following sections we highlight some key mutations detected in ABC carbohydrate transport systems and sensor kinases.

### Mutations in the ABC Transport Complex Substrate-binding Protein Cphy 2654

ABC carbohydrate transporters consist of an extracellular substrate-binding protein, a membrane spanning permease and an ATPase that drives carbohydrate transport. In the xylan-adapted lines, two different lines (A and C), had mutations in the same substrate-binding protein (Cphy 2654). In Xyn-A, a G457E mutation was detected in 90% of the population. The Xyn-C population had three alleles at the same site in this gene. A Y196S substitution was present in 43% of the population while 16% displayed a Y196N mutation.

**Figure 6:**
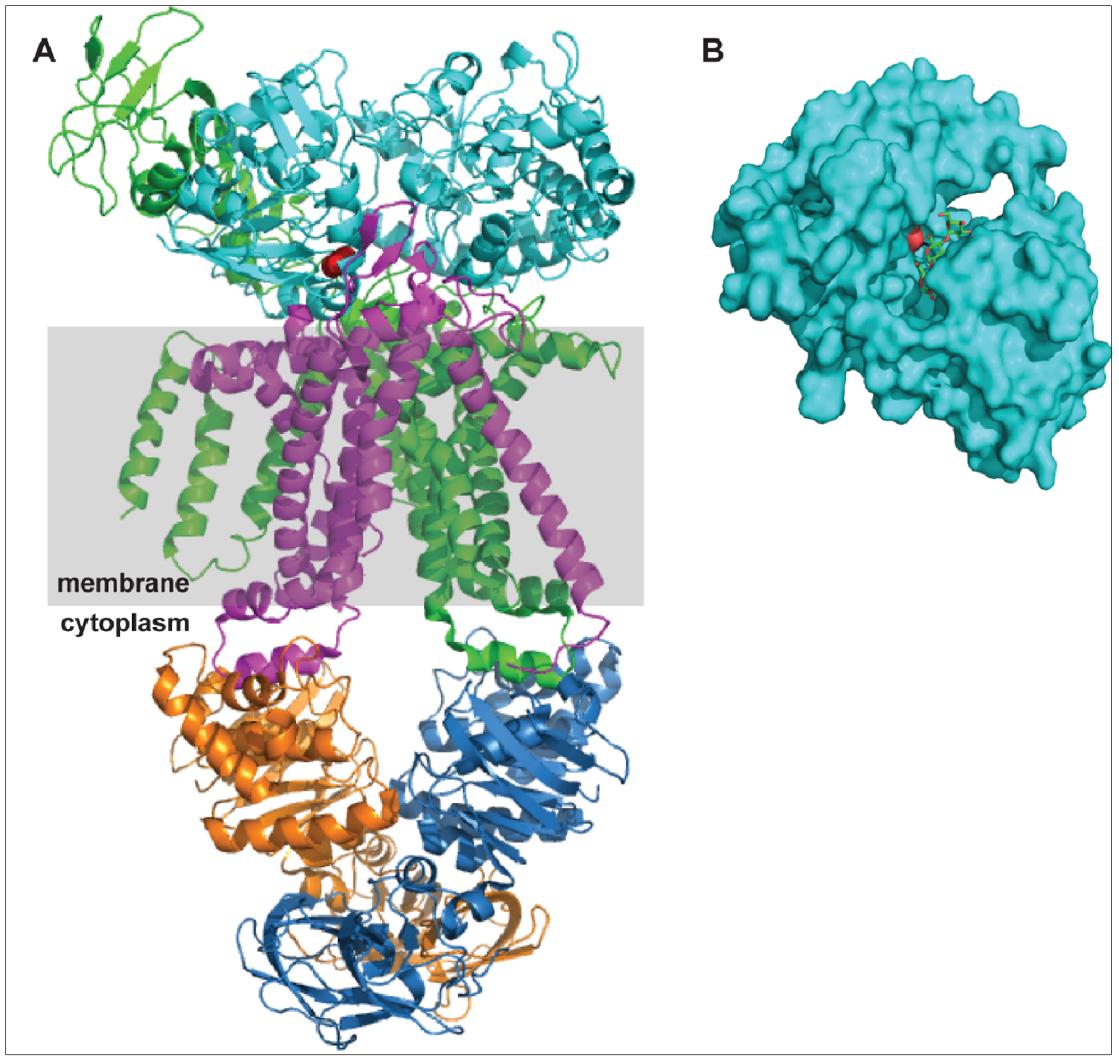
Homology models suggest that the selected mutations in an ABC transporter binding protein occur at protein-protein and protein-ligand interfaces. Three mutations in Cphy 2654 (G457E, Y196N, and Y196S) were found in xylan-adapted populations. A. The structure of the maltose transporter complex with maltose binding protein (3pv0) is shown with the maltose binding protein replaced by a homology model of Cphy 2654 (cyan cartoon, based on 3omb). The model suggests that the Cphy 2654 G457E mutation (red) is near the interface between the binding protein and the transmembrane domains. B. Surface representation of another homology model of Cphy 2654 (based on 2fnc) shows that the Y196N/S mutations (red) is predicted to occur in the ligand binding pocket.

We used homology modeling to interpret the location within the likely protein structure of the G457E and Y196S/N. The SWISS-Model [42] server in automatic mode on the full-length *C. phytofermentans* protein produced a single structural model based on the template 3omb (an extracellular binding protein with no ligand in the structure). To interpret the location of the G457E mutation within a transporter complex, the *C. phytofermentans* structural model (residues 69–586) was aligned to the *E. coli* maltose binding protein within the full maltose transporter complex. Using the cealign [43] command in Pymol, this alignment was done with the various available maltose transporter structures to determine that 3pv0 gave the best alignment (lowest rmsd over the largest number of amino acids) with the *C. phytofermentans* model. Figure 6A shows the alignment of the *C. phytofermentans* model to the maltose binding protein (chain E) of the transporter complex (3pv0) and reveals that the G457E mutation (red) is likely to be near the interface of the binding protein (cyan) with one of the transmembrane domains (MalG, magenta).

We performed additional modeling with the goal of constructing a model with a template that included an oligosaccharide ligand. The N-terminal truncated Cphy 2654 sequence (missing the first 30 amino acid transmembrane anchor) submitted to the SWISS-model server in automatic mode yielded a good candidate: 2fnc, one of the 3 selected templates, is a maltotriose binding protein with maltotriose bound. Figure 6B shows the *C. phytofermentans* model (cyan surface) with the 2fnc ligand (stick model) in the pocket. The side chain oxygen of the *C. phytofermentans* mutation site, Y196 (red), is visible within the binding pocket and is within hydrogen bonding distance of a ligand oxygen. Another model was constructed using the specified template 2z8f, which was template #3 in the HHSearch of SWISS-model template identification and corresponds to a binding protein complex with lacto N tetraose. Again the Y196 mutation site borders on the ligand pocket, though it is not as close to the ligand as in the 2fnc-based model structure. Of course we do not know the native ligand of Cphy 2654, but models constructed with template binding proteins of oligosaccharides with either alpha(1→4) or beta(1→4) linkages (maltotriose and lactoNtetraose, respectively) both suggest that the Y196 mutation occurs in the ligand pocket.

### Mutations in the ABC Transporter Membrane Permease Cphy 2465

**Figure 7:**
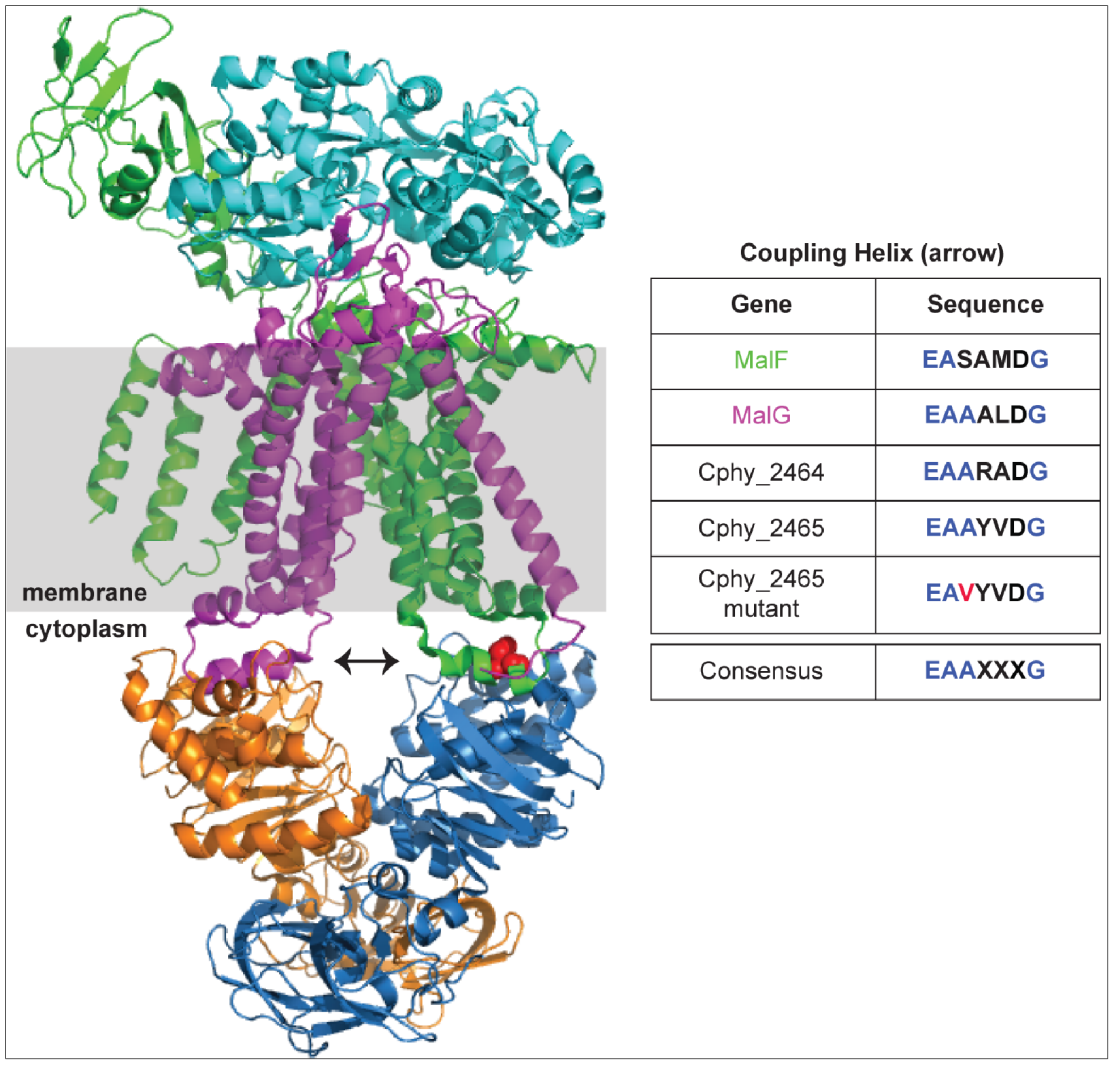
Homology modeling suggests that a selected mutation in an ABC transporter transmembrane domain (Cphy 2465) in cellulose-adapted populations occurs at a protein-protein interface. The maltose transporter (3pv0) is shown because its transmembrane domains MalF (green) and MalG (magenta) are the best templates of known structure for Cphy 2465 and Cphy 2464, respectively. A homology model of Cphy 2465 based on MalF places the selected A207V mutation (red) in the coupling helix (arrow and table) that is important in transmitting changes between the transmembrane domains (green and magenta) and the ATPase domains (blue and gold). The mutation occurs in the consensus sequence originally identified in several transporters [45].

A portion of Cel-A population acquired a SNP in Cphy 2465, the permease component of an ABC transporter. To determine the location of the mutation on the complete protein and predict its possible role in facilitating substrate transport in the adapted lines, we created homology models of Cphy 2465 based on known crystal structures of the *E. coli* MalFGK2 transporter system. Maltose transporter transmembrane domains MalF (green in Fig. 7) and MalG (magenta in Fig. 7) are the best templates of known structure for Cphy 2465 and Cphy 2464, respectively. The A207V mutation selected in Cphy 2465 during growth on cellulose occurs in the “coupling helix” (arrow and table in Fig. 7), which is thought to be important in coupling ATP hydrolysis catalyzed by the ATPase domains (blue and gold in Fig. 7) to transport by the transmembrane domains (magenta and green in Fig. 7). This coupling helix is present in all known structures of ABC transporters [44]. Although early analysis of several transporters identified a consensus sequence [45] that is also present in the Cphy 2464–2465 transporter domains (Fig. 7), comparison of a larger number of transporters indicates greater sequence variability, which may be important in conferring specificity at this protein-protein interface [46]. The mutation site is shown in red both in the structure of MalF (Fig. 7) and in the original consensus sequence identified for the coupling helix (Fig. 7).

### Sensor Kinase Mutations

**Figure 8:**
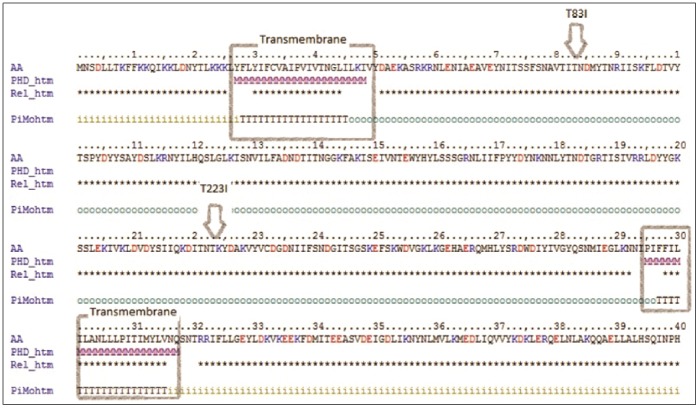
Localization of SNPs in Cphy 3212 cellulose adapted lines. T83I in Cel-C and T223I in Cel-B. Predicted transmembrane regions of the protein are highlighted with a grey box, strings of ‘o’ represent the extracytoplasmic regions, while the regions marked ‘i’ are predicted to lie within the cell. Both SNPs are located in the region of the protein predicted to be on the extracellular face.

Mutations were identified in two different histidine kinase genes. Ceb-C populations acquired a single deletion in Cphy 0155 which is predicted to be a signal transduction sensor histidine kinase. The deletion would cause a frameshift at the beginning of the histidine kinase protein and is fixed in the population. Ceb-B and Ceb-C populations accumulated two independent threonine to isoleucine mutations (T83I & T223I) in Cphy 3212 which is annotated to be a part of a two component sensor histidine kinase system located upstream of an AraC type transcriptional regulator (Cphy 3211). Cphy 3212 and Cphy 3211 are adjacent to an ABC transporter operon which has been observed to be highly expressed in founder cells grown on cellulose (Petit et al. submitted). We used the PredictProtein [47] sequence analysis server to determine the localization of the two SNPs within the protein. Cphy 3212 was predicted to have an extracytoplasmic domain flanked by two short transmembrane domains and a C-terminal cytoplasmic kinase domain. Both SNPs were predicted to lie in the extracytoplasmic region between the membrane spanning domains (Fig. 8).

## Discussion

We have demonstrated that: (1) Repeated serial transfer during exponential growth on plant cellobiose, cellulose and xylan selects for populations with higher rates of carbohydrate utilization and product formation. (2) High throughput sequencing of the evolved populations can be used to reliably identify polymorphisms at frequencies greater than 5%. (3) Many mutations are in coding or regulatory regions of genes related to carbohydrate usage. (4) Structural modeling of mutations in ABC transporter components suggest changes in substrate recognition and transport.

### High throughput Sequencing of the Evolved Populations

To date efforts to identify molecular changes in evolved populations involved isolating and analyzing individual clones as representatives of an adapted population. Recent improvements in genome sequencing technology and subsequent analysis pipelines encouraged us to take a broader approach and sequence entire populations to assay genetic variation, before deciding whether to isolate individual clones. Our population-level mutation detection protocol involved a combination of automated tools and manual evaluation which identified several mutations within each population at various levels of fixation.

The fact that the adapted populations share few parallel mutations even when adapted under similar conditions suggests that there can be multiple paths to attain a desired fitness peak within a population. This is consistent with previous ideas that replicate populations from the same founder can undertake separate fitness trajectories and attain different adaptive peaks even if evolved in similar environments [48]. Further studies involving a temporal analyses of the adapted populations are needed to follow the trajectory and determine the step wise process of evolution in these *C. phytofermentans* lines.

### IS Element Mutations

The cellulose adapted lines, especially Cel-B and Cel-C show a high level of insertion sequence (IS) element activity (Table S1). Insertion sequences are transposable elements with short inverted repeat sequences which are sites for transposition by transposases. Insertion elements have been shown to be a contributing factor shaping evolution in microbes by creating genomic rearrangements through insertion, deletion and gene duplication. Genome evolution in *Mycobacterium smegmatis* was shown to be largely due to IS1096 type insertion elements [49]. IS204-type insertion elements, first reported in *Nocardia asteroides* bear high sequence similarity with IS1096 sequences. IS204 mediated mutagenesis was carried out in-vitro in *Streptomyces coelicolor* using plasmids constructed from IS204 elements [50]. The *C. phytofermentans* genome contains insertion elements belonging to the IS204/IS1001/IS1096/IS1165 family. Several insertions by elements in this family appeared in our cellulose evolved lines. However, the functional consequences of these transpositions was not apparent. It is plausible that expression of these elements is condition-specific as they were observed only in cellulose-adapted populations.

### Higher Rates of Carbohydrate Utilization and Product Formation

Cellobiose and xylan-adapted populations had higher growth yields as measured by final optical density compared to their founder, increased growth rates and produced ethanol faster. These results are similar to others studies in *E. coli* and *S. cerevisiae* in which growth rate and growth yield increased simultaneously [51]–[53].

When *C. phytofermentans* is grown on cellobiose and xylan, the corresponding hydrolases and ABC transport systems are expressed at very high levels (Petit et al, submitted). Thus, we did not expect to see further increase in expression of respective carbohydrate breakdown genes in our adapted populations. This was confirmed by microarray analysis of the cellobiose-adapted populations, which did not show a significant increase in gene expression of the cellobiose ABC transport complex genes (Cphy 2464, 2465 and 2466) or the cellobiose phosphorylase (Cphy 0430) relative to the founder (Table S2).

### Many Mutations are in Coding or Regulatory Regions of Genes Related to Carbohydrate Usage

ABC carbohydrate transport systems have been classified into two categories based on an ATPase activity that is either fused to the permease (CUT2-type) or found as a separate intracellular protein (CUT1-type). The CUT1-type are involved in oligosaccharide transport while the CUT2-type are responsible for monosaccharide transport [54]. All mutations identified in our experiment are in the protein or regulatory regions of CUT1-type transporters, suggesting that these mutations effect oligosaccharide transport. Co-evolution of ABC transport systems along with regulatory sensor kinases has been widely observed in firmicutes, especially in *Bacilliales* and *Clostridiales* where multiple such interacting components are present [55]. The histdine kinase Cphy 3212 mutated in Cel-B and Cel-C is adjacent to an ABC transporter operon which has been observed to be highly expressed in founder cells grown on cellulose (Petit et al. submitted). The transporter system and sensor kinase genes may not necessarily be a part of the same operon system to form a regulatory partnership. Histidine kinase genes from separate genetic loci have been shown to regulate expression of ABC transport system in *Listeria monocytogenes* and *Staphylocuccus aureus* [56], [57]. The histidine kinase Cphy 0155 is in an operon with a response regulator, but is not adjacent to a transport system, and thus it is not clear from the expression data what functional role it plays.

There were several other mutations in protein coding regions of potential significance. Cphy 0515, which is annotated as a hypothetical protein, lies on an operon with a RNA polymerase sigma factor subunit. This gene was under very strong selection in Xyn-B population as it acquired 3 independent mutations within 12 bp of each other. All three of the mutations, a non-synonymous substitution, a single bp insertion and a 5 bp deletion result in a truncated version of the protein. Sigma factors often regulate environmental specific responses, but the role of this specific sigma factor in *C. phytofermentans* is unknown.

**Figure 9:**
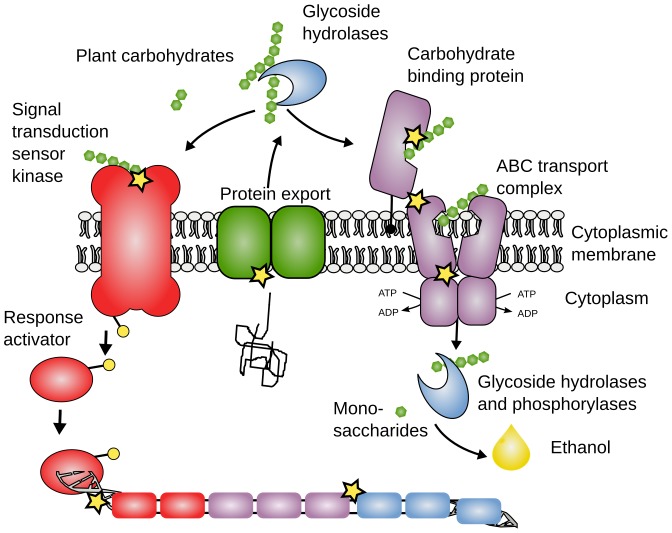
Overview of carbohydrate sensing, saccharification and transport systems with the approximate location of evolved mutations. The mutations are represented by yellow stars.

Cphy 2284 is annotated as a signal peptidase which under normal growth conditions is highly expressed on all the substrates assayed in our microarray experiments (Petit el al in preparation). 32% of the Xyn-C population has a non-synonymous substitution from isoleucine to lysine in Cphy 2284. We speculate that this mutation may allow for faster export of glycoside hydrolases and other extracellular proteins. A diagrammatic representation of this and other evolved mutations is shown in Figure 9.

### Mechanistic Insights into ABC Transport System Function

One of the best characterized ABC transport systems is *E. coli* maltose transporter MalFGK2. Homology modeling was performed on the evolved genes Cphy 2465, the membrane-spanning permease component and Cphy 2654, the substrate-binding part of ABC transporters. The *C. phytofermentans* multifunctional ATPase (Cphy 3611) shares more than 50% sequence identity with *E. coli* MalK while the membrane permease domains share around 20% identity with *E. coli* MalF and MalG. Despite the low sequence conservation of the permease domain, which is typical for ABC transporters [58]–[61], the Type I ABC importers of known structure (maltose, molybdate, and methionine) have permease domain core helix backbone structures that are superimposable with a 2.5 Å rmsd [58].

Our modeling studies identified mutations located at interfaces between the protein components of ABC transport systems. The structural model for Cphy 2465 predicts a possible mechanism whereby a mutation in the coupling helix could very well lead to improved interaction between the permease domain and multifunctional ATPase domain. The likely locations of the Cphy 2654 mutations based on homology models suggest that the mutations could alter the interactions of this binding protein with its ligand and with the transmembrane domains of the transporter complex. These mutations suggest that improvements in transporter efficiency for new substrates can be generated in different ways involving the coupling between the different transport components, and not just at the ligand binding step. This is analogous to molecular genetics and biochemical studies of the maltose permease in which compensatory mutations following the deletion of the binding protein resulted in uptake of maltose to support growth [62]. These mutations led to constitutive ATPase activity in the maltose transporter suggesting that mutations perturbing the coupling between components can allow substrate uptake under conditions where it otherwise would not occur [63], [64]. Additional experiments along these lines will improve our mechanistic understanding of ABC transporters which can then be engineered to transport plant carbohydrates with increased efficiency.

## Conclusion

To our knowledge, this is the first laboratory adaptive evolution experiment with a cellulosic microbe on the primary components of plant cell wall hemicellulose and cellulose. The adapted populations displayed increased growth rates and ethanol production capabilities compared to their founder. Genome resequencing identified mutations in carbohydrate-related pathways in the adapted lines, which may play an important role in overcoming constraints on carbohydrate uptake and transport in *C. phytofermentans*. Although plant biomass is widely available, the cost of degrading the hemicellulosic and cellulosic portions of the plant cell wall currently is a major limiting factor in many applications such as biofuels and livestock feed conversion. Novel strains of a microbe, which in isolation or as a consortium of closely related strains breaks down plant biomass would improve industry economics. Future characterization of the mutations identified in our study will help better understand mechanisms involved in the bacterial decomposition of plant cell walls.

## Methods

### Organism and Medium

An isogenic colony of *C. phytofermentans* strain ISDg was used as the founder of our adaptive evolution experiments. This strain has been deposited at the American Tissue Culture Collection and the genome sequence is available in GenBank (NC_010001). To obtain an isogenic strain of the ancestor, cells were taken out of the freezer and plated on modified GS-2 agar medium [37] supplemented with 0.6% cellobiose for 6 days to obtain isolated colonies. A single colony was transferred to modified GS-2 medium supplemented with 0.3% cellobiose for 48 hours and stored at −80°C in 15% glycerol. Nine individual populations, 3 in each substrate, were initiated from the founder strain and evolved separately on cellobiose (Ceb-A, Ceb-B & Ceb-C), cellulose (Cel-A, Cel-B & Cel-C) and xylan (Xyn-A, Xyn-B & Xyn-C). We used commercially available cellobiose and xylan (Sigma) while the cellulose for our experiment was #1 Whatman filter paper cut into small pieces. The filter paper pieces were pebble milled for 7 days with distilled water to make a 3% slurry and autoclaved for 20 minutes. This slurry was added to GS-2 tubes to make a final concentration of 0.6% (wt/vol). For each carbon substrate, three replicate lines were established from the founder strain. Cells were grown anaerobically at 30°C under conditions of 100% nitrogen in 20 ml culture tubes with 10 ml modified GS-2 medium supplemented with 0.3% (wt/vol) concentration of the specific carbon source. In addition to carbon, the GS-2 medium includes KH_2_PO_4_, Na_2_HPO_4_, urea, cysteine HCl, sodium citrate, yeast extract and resazurin at defined concentrations.

### Serial Transfers

For each transfer, 200 µl of the previous culture was moved to 10 ml of fresh medium. Transfer times were established based on the expected time cells would reach mid to late exponential phase and to allow for transfer periods in multiples of 24 hours. These transfer times were every 24 hours on xylan, 48 hours on cellobiose and every 7 days on cellulose. Periodically, cultures were checked for contamination using phase contrast microscopes, streaking on the surface of agar and using a customized PCR assay developed specifically for detecting contaminants growing with *C. phytofermentans*. The principle of the contamination detection assay is based on ribosomal intergenic spacer analysis [65], [66] which involves PCR amplification of the spacer region between the 16S and 23S genes of the rRNA locus.

### Analytical Procedures

Growth on soluble substrates (cellobiose and xylan) was determined spectrophotometrically by monitoring changes in optical density at 660 nm. Comparisons of cellulose adapted lines to the founder were done by visual examination of the amount of cellulose remaining in the culture tubes and using liquid fermentation products as a proxy of growth. Non-gaseous fermentation products were determined by high performance liquid chromatography (HPLC). A culture sample (10 ml) was centrifuged at 4000 rpm for 30 minutes at 4°C and 1 ml aliquots of the supernatant were stored at −20°C for subsequent analysis. Ethanol, acetate, lactate and formate concentrations were measured using a BioRad Aminex HPX 87 H 300×7.8 mm column with 0.005 M H_2_SO_4_ as the running buffer in a Hitachi model L-7100 HPLC unit equipped with a Sonntek Refractive Index Detector.

### Genomic DNA Extraction and Whole Genome Sequencing

Whole genome sequencing of the founder and adapted lines were performed using Illumina Sequencing technology. High quality genomic DNA was extracted from mid to late exponential phase cultures using a CTAB-based extraction protocol. Concentration and integrity of the isolated DNA was confirmed by gel electrophoresis including the Joint Genome Institute DNA Mass Standards. DNA was sheared into ∼230 bp fragments and the resulting fragments were used to create an Illumina sequencing library. These libraries were sequenced on Illumina HiSeq generating an average of 13,600,000 paired end reads (100 bp) per population. The sequence data has been deposited in the NCBI sequence read archive under accession number SRP02917.

### Confirmation of Select Mutations

We applied traditional Sanger sequencing of selected loci containing mutations to confirm the frequency of two independent SNPs in Cphy 2654. A 250–300 bp region surrounding the SNP was amplified using custom primers and sequenced. Direct inspection of sequence chromatogram was carried out using Sequence Scanner v1.0 (Life Technologies Corp., Carlsbad, CA). The height of the sequencing chromatogram was used to determine the frequency of the dominant nucleotide in a particular population. Illumina sequencing and subsequent analysis methods predicted a G457E SNP (GGA -> GAA; Figure S1) in Cphy 2654 (Table S1) to be present in 90% of Xyn-A lines while a Y587S SNP (TAT -> TA/CT; Figure S1) was predicted in approximately 43% of Xyn-C lines. The sequencing chromatogram from PCR products obtained by amplifying regions around the respective mutations supports the above frequency values (Figure S1).

### RNA Isolation and Gene Expression Analysis of Cellobiose-adapted Lines

RNA was isolated from mid-exponential phase cultures on GS-2 medium supplemented with 0.3% cellobiose. 1 ml samples were taken and flash-frozen by immersion of the tubes in liquid nitrogen. The cells were centrifuged for 5 minutes at 8,000 rpm at 4°C, and the total RNA was isolated using Trizol followed by the Qiagen RNeasy Mini Kit according to the manufacturer’s instructions. The RNA concentration was determined by absorbance at 260/280 nm using a Nanodrop spectrophotometer and RNA integrity was checked using an agarose gel. Subsequent steps like cDNA synthesis, array hybridization and imaging were performed at the Genomic Core Facility at the UMass Medical Center. The raw microarray data sets were normalized using Robust Multi-Array average (RMA) implemented in BioConductor [67]. The normalized expression files were analyzed using the Multiexperiment Viewer (MeV) [68]. To help identify similarities and differences in the expression profiles among the evolved and the parental strains, the gene expression patterns were subjected to cluster analyses implemented within MeV. The calculated expression values of Ceb-A, Ceb-B, Ceb-C & Ceb-F are listed in Table S2. The microarray data and.CEL files have been deposited in NCBI’s GEO database under accession number GSE52494.

## Supporting Information

Figure S1 **Frequency of SNPs confirmed using Sanger sequencing.**
(TIF)

Table S1 **Complete list of mutations identified in the adapted populations.**
(PDF)

Table S2 **Gene expression values of cellobiose adapted populations and the founder.**
(XLS)
