# Supplementary figures and images for: Population Level Analysis of Evolved Mutations Underlying Improvements in Plant Hemicellulose and Cellulose Fermentation by Clostridium phytofermentans
Source: PLoS One. 2014 Jan 22;9(1):e86731. doi: 10.1371/journal.pone.0086731 (PMC3899296; doi:10.1371/journal.pone.0086731)

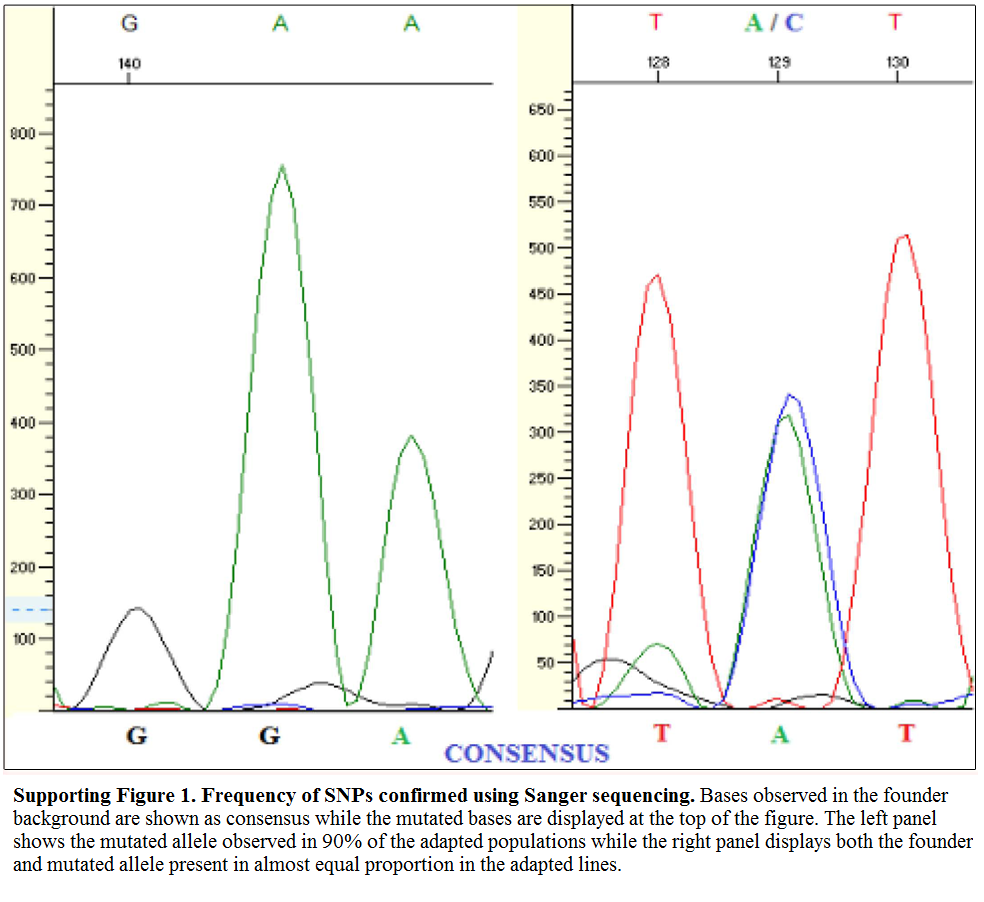

Supplement: Figure S1 — Frequency of SNPs confirmed using Sanger sequencing. (TIF) [file pone.0086731.s001.tif]
