# Supplementary material for: Population Level Analysis of Evolved Mutations Underlying Improvements in Plant Hemicellulose and Cellulose Fermentation by Clostridium phytofermentans
Source: PLoS One. 2014 Jan 22;9(1):e86731. doi: 10.1371/journal.pone.0086731 (PMC3899296; doi:10.1371/journal.pone.0086731)
Supplement: Table S1 — Complete list of mutations identified in the adapted populations. (PDF) [file pone.0086731.s002.pdf]

Population	Mutation Type	Genomic Attribute	Change	Genome Location	Gene	Function	Role	Frequency
Ceb-A	Insertion	IVS	+1bp	4051009**	Cphy_3328	hypothetical protein		100%
					Cphy_3329	Beta-glucosidase; GH family 3 domain protein	Hydrolase	
	SNP	CDS	G201R	4421212	Cphy_3580	protein of unknown function DUF181		15%
	SNP	CDS	D156V	4836776	Cphy_3936	transcriptional regulator, LuxR family	Regulation	4%
Ceb-B	Insertion	CDS	+1bp	656339	Cphy_0515	hypothetical protein		100%
	Deletion	IVS	-194bp	4488958	Cphy_3637	ATPase component of ABC transporter	Transport	100%
					Cphy_3638	ribosomal protein	Regulation	
	Deletion	CDS	-194kbp	2486444	Cphy_2013	proteinase inhibitor I4 serpin		50%
Ceb-C	Insertion	IVS	+1bp	4051009**	Cphy_3328	hypothetical protein		100%
					Cphy_3329	Beta-glucosidase; GH family 3 domain protein	Hydrolase	
	Deletion	CDS	-1bp	200796	Cphy_0155	signal transduction histidine kinase	Regulation	100%
	Deletion	CDS	-10bp	2886533	Cphy_2341	alpha amylase catalytic region	Hydrolase	91%
	SNP	CDS	K463Q	1248484	Cphy_0981	hypothetical protein		13%
Xyn-A	Deletion	IVS	-56bp	3512573	Cphy_2862	signal transduction histidine kinase	Regulation	100%
					Cphy_2863	DNA-binding domain protein	Regulation	
	Insertion	IVS	+1bp	4051009	Cphy_3328	hypothetical protein		100%
					Cphy_3329	Beta-glucosidase; GH family 3 domain protein	Hydrolase	
	SNP	CDS	G457E	3240019	Cphy_2654	ABC transporter substrate-binding protein	Transport	90%
Xyn-B	Deletion	IVS	-56bp	3512573	Cphy_2862	signal transduction histidine kinase	Regulation	100%
					Cphy_2863	DNA-binding domain protein	Regulation	
	Deletion	CDS	-5bp	656349	Cphy_0515	hypothetical protein		40%
	Insertion	CDS	+1bp	656337	Cphy_0515	hypothetical protein		30%
	SNP	CDS	I32K	2818290	Cphy_2284	signal peptidase I	Export	32%
	SNP	CDS	K35Stop	656338	Cphy_0515	hypothetical protein		30%
	SNP	CDS	Q88Stop	156629	Cphy_0116	transcriptional regulator	Regulation	5%
	Deletion	IVS	-56bp	3512573	Cphy_2862	signal transduction histidine kinase	Regulation	100%
					Cphy_2863	DNA-binding domain protein	Regulation	

Xyn-C	Insertion	IVS	+1 bp	4051010	Cphy_3328	hypothetical protein		100%
					Cphy_3329	Beta-glucosidase; GH family 3 domain protein	Hydrolase	
	SNP	CDS	Y196S	3240802	Cphy_2654	ABC transporter substrate-binding protein	Transport	43%
	SNP	CDS	Y196N	3240803	Cphy_2654	ABC transporter substrate-binding protein	Transport	16%
Cel-A	SNP	CDS	P193S	3270649***	Cphy_2680	ribosomal protein S2	Translation	100%
	Insertion	IVS	+1bp	4051009***	Cphy_3328	hypothetical protein		100%
					Cphy_3329	GH family 3 domain protein	Hydrolase	
	Deletion	IVS	+2bp	1646255	Cphy_1314	Transposase		80%
					Cphy_1315	Electron transfer flavoprotein subunit		
	Insertion	IVS	-2bp	1644821	Cphy_1313	Transcription regulator	Regulation/IS	75%
					Cphy_1314	Transposase		
	SNP	CDS	Q1043Stop	2322775	Cphy_1882	Parallel beta-helix repeat	Regulation	50%
	Deletion	CDS	-19kbp	2491870	Cphy_2018	transcriptional regulator	Regulation	42%
	IS Insertion	IVS	IS	3508335	Cphy_2858	Thymidine kinase		40%
					Cphy_2859	Transcription regulator	Regulation	
	SNP	CDS	Q101Stop	1587995	Cphy_1257	Extracellular ligand-binding receptor		30%
	SNP	CDS	T96M	4476057	Cphy_3624	hypothetical protein		25%
	SNP	CDS	P168S	3611513	Cphy_2939	band 7 protein		20%
	SNP	IVS	A207V	3026481	Cphy_2465	Binding-protein-dependent transport systems	Transport	13%
	SNP	CDS	D200N	3960794	Cphy_3256	transcriptional regulator, AraC family	Translation	6%
	SNP	IVS	NC	4110150	Cphy_3368	Cellulose 1,4-beta-cellobiosidase	Hydrolase	5%
					Cphy_3369	heat shock protein Hsp91		
	SNP	IVS	NC	1836285**	Cphy_1492	YheO domain protein		100%
					Cphy_1493	endoribonuclease L-PSP		
	SNP	CDS	P193S	3270649***	Cphy_2680**	ribosomal protein S2	Translation	100%
	IS insertion	IVS	IS	2329627	Cphy_1886	N-acetylmuramoyl-L-alanine amidase		100%
					Cphy_1887	putative ATP-binding protein		
	IS insertion	IVS	IS	3004929**	Cphy_2447	fatty acid/phospholipid synthesis protein PlsX		100%

	insertion				Cphy_2448	MCP methyltransferase, CheR-type	Regulation	
	IS insertion	IVS	IS	3508296**	Cphy_2858	Thymidine kinase		100%
					Cphy_2859	helix-turn-helix domain protein	Regulation	
	Insertion	IVS	+1bp	4051009***	Cphy_3328	hypothetical protein		100%
					Cphy_3329	Beta-glucosidase; GH family 3 domain protein	Hydrolase	
Cel-B	IS insertion	CDS	IS	4317059**	Cphy_3499	polysaccharide biosynthesis protein		100%
	IS insertion	IVS	IS	4331228**	Cphy_3509	Ig domain protein group 2 domain protein		100%
					Cphy_3510	hypothetical protein		
	IS insertion	IVS	IS	4824521**	Cphy_3925	iron-containing alcohol dehydrogenase		100%
					Cphy_3926	ABC transporter related	Transport	
	Deletion	IVS	-5bp	1552121**	Cphy_1228	Pyridoxal-5'-phosphate-dependent protein beta subunit		37%
					Cphy_1229	transcriptional regulator, MarR family	Regulation	
	Insertion	IVS	+6bp	1644823**	Cphy_1313	regulatory protein GntR HTH	Regulation/IS	33%
					Cphy_1314	Transposase		
	Deletion	IVS	-6bp	1646255**	Cphy_1314	Transposase		29%
					Cphy_1315	Electron transfer flavoprotein subunit		
	SNP	CDS	T668I	3918309	Cphy_3212	histidine kinase internal region	Regulation	16%
	SNP	IVS	NC	1836285**	Cphy_1492	YheO domain protein		100%
					Cphy_1493	endoribonuclease L-PSP		
	SNP	CDS	P193S	3270649***	Cphy_2680	ribosomal protein S2	Translation	100%
	IS insertion	IVS	IS	3004929**	Cphy_2447	fatty acid/phospholipid synthesis protein PlsX		100%
					Cphy_2448	MCP methyltransferase, CheR-type		
	IS insertion	IVS	IS	3508296**	Cphy_2858	Thymidine kinase		100%
					Cphy_2859	helix-turn-helix domain protein	Regulation	
	Insertion	IVS	+1bp	4051009***	Cphy_3328	hypothetical protein		100%
					Cphy_3329	GH family 3 domain protein	Hydrolase	
	IS insertion	CDS	IS	4317059**	Cphy_3499	polysaccharide biosynthesis protein		100%

Cel-C	IS insertion	IVS	IS	4331228**	Cphy_3509	Ig domain protein group 2 domain protein		100%
					Cphy_3510	hypothetical protein		
	IS insertion	IVS	IS	4824521**	Cphy_3925	iron-containing alcohol dehydrogenase		100%
					Cphy_3926	ABC transporter related	Transport	
	Deletion	IVS	-5bp	1552121**	Cphy_1228	Pyridoxal-5'-phosphate-dependent protein beta subunit		38%
					Cphy_1229	transcriptional regulator, MarR family	Regulation	
	SNP	IVS	NC	4110124	Cphy_3368	Cellulose 1,4-beta-cellobiosidase	Hydrolase	32%
					Cphy_3369	heat shock protein Hsp90		
	Insertion	IVS	+6bp	1644823**	Cphy_1313	regulatory protein GntR HTH	Regulation/IS	20%
					Cphy_1314	Transposase		
	Deletion	IVS	-6bp	1646255**	Cphy_1314	Transposase		20%
					Cphy_1315	Electron transfer flavoprotein subunit		
	Deletion	IVS	-1bp	2869121	Cphy_2328	Transposase		17%
					Cphy_2329	Deoxyadenosine kinase		
	SNP	CDS	T8931	3918729	Cphy_3212	histidine kinase internal region	Regulation	13%
	SNP	CDS	I425I	770792	Cphy_0592	extracellular solute-binding protein family 1	Transport	9%
	SNP	CDS	Stop104Sto	4240411	Cphy_3448	hypothetical protein		8%
	SNP	CDS	I25K	1103738	Cphy_0863	histidine kinase internal region	Regulation	7%
Note: Frequency reported for indels is a rough estimate calculated by counting the number of reads that showed the indel vs the number which did not.								
*** Present in all three lines								
** Present in two lines								
CDS: Coding DNA Sequence								
IVS: Intervening Sequence								
Frequency of several identical mutations in Cel-B and Cel-C suggest a possibility of the populations being swapped due to experimental error.								
